# Benefits and Risks Associated With Aspirin Use in Patients With Diabetes for the Primary Prevention of Cardiovascular Events and Mortality: A Meta-Analysis

**DOI:** 10.3389/fendo.2021.741374

**Published:** 2021-09-01

**Authors:** Hua Ma, Qing Gu, Huining Niu, Xiaohua Li, Rong Wang

**Affiliations:** ^1^ Deparment of Vasculocardiology, Xianyang Central Hospital, Xianyang, China; ^2^ Deparment of Vasculocardiology, Peking Union Medical College Hospital, Chinese Academy of Medical Sciences, Beijing, China; ^3^ Department of Hematology, Xianyang Central Hospital, Xianyang, China; ^4^ Department of General Surgery, The Second People’s Hospital, Kunshan, Suzhou, China

**Keywords:** aspirin, diabetes, cardiovascular diseases, mortality rate, MACE

## Abstract

**Purpose:**

A meta-analysis was conducted to assess the benefits and risks of aspirin for the primary prevention of cardiovascular disease and all-cause mortality events in adults with diabetes.

**Methods:**

An extensive and systematic search was conducted in MEDLINE (via PubMed), Cinahl (via Ebsco), Scopus, and Web of Sciences from 1988 to December 2020. A detailed literature search was conducted using aspirin, cardiovascular disease (CVD), diabetes, and efficacy to identify trials of patients with diabetes who received aspirin for primary prevention of CVD. Demographic details with the primary outcome of events and bleeding outcomes were analyzed. The Cochrane Collaboration’s risk of bias tool was used to assess the methodological quality of the included studies. Random-effects meta-analysis was used to calculate the pooled odds ratio for outcomes of cardiovascular events, death, and adverse events.

**Findings:**

A total of 8 studies were included with 32,024 patients with diabetes; 16,001 allocated to aspirin, and 16,023 allocated to the control group. There was no difference between aspirin and control groups with respect to all-cause mortality, cardiovascular mortality, or bleeding events. However, MACE was significantly lower in the aspirin group.

**Implications:**

Although aspirin has no significant risk on primary endpoints of cardiovascular events and bleeding outcomes in patients with diabetes compared to control, major adverse cardiovascular events (MACE) were significantly lower in the aspirin group. Further research on the use of aspirin alone or in combination with other antiplatelet drugs is required in patients with diabetes to supplement currently available research.

**Systematic Review Registration:**

identifier [XU#/IRB/2020/1005].

## Highlights

What is already known?

Cardiovascular disease (CVD) is one of the primary causes of mortality in people with diabetes, accounting for >70% of deaths in these people. Low-dose aspirin is a widely used agent for preventing cardiovascular disease, and various guidelines also recommend its use in subjects with diabetes. Several primary prevention aspirin trials- prior to 2000 suggested a reduction in myocardial infarction and stroke, although not mortality but at the cost of increased bleeding events. However, the role of aspirin in the primary prevention of CVD is still -debatable.

What is new?

- The present meta-analysis provides an insight into the current use of aspirin in reducing the risk of cardiovascular events in patients with diabetes.
- Furthermore, a new study horizon is needed to evaluate further the risks associated with aspirin plus newer antiplatelet drugs.

## Introduction

Diabetes results in a two- to four-fold higher risk of cardiovascular diseases (1). In addition, cardiovascular disease (CVD) is the primary cause of death among persons with diabetes, accounting for more than 70% of fatalities (2). As a result, there has been a surge in interest in developing therapies to lower cardiovascular risk in patients with diabetes in recent decades.

Several anomalies in platelet function have been found in -patients with diabetes (3), leading to an accelerated state of atherosclerosis and inflammation, which increases vascular problems.Therapies that block platelet activation and aggregation, such as aspirin treatment, have been advocated as critical therapeutic methods for lowering ischemic risk in diabetics (4). Low-dose aspirin has been used to treat and prevent cardiovascular disease for decades. In 2007, the American Diabetes Association (ADA) and the American Heart Association (AHA) jointly recommended that aspirin therapy (75-162 mg/day) be used as a primary prevention strategy for those with diabetes at increased cardiovascular risk, including those who are over 40 years of age or have additional risk factors (family history of CVD, hypertension, smoking, dyslipidemia or albuminuria) (1, 3).

The efficacy of aspirin in the secondary prevention of CVD in patients with diabetes has been well demonstrated (5). The function of aspirin in the primary prevention of CVD in persons with diabetes has been studied in several randomized controlled trials (6–13). However, most of these studies were underpowered in terms of the number of persons with diabetes, reported data from subgroups, and reported inconsistent results. Since the Antithrombotic Treatment Trialists’ Collaboration published a meta-analysis of individual-level data from six primary prevention trials in 2009, which reported a nonsignificant reduction in serious vascular events in people with diabetes (14), several further meta-analyses have been conducted on the topic, with no significant benefit for aspirin in the primary prevention setting (13, 15–20).

Recent guidelines from the Fifth Joint Task Force of the European Society of Cardiology and Other Societies on CVD Prevention in Clinical Practice do not give explicit recommendations for aspirin in patients with diabetes, which is consistent with the ambiguous data (11). The American Diabetes Association, the American Heart Association, and the American College of Cardiology Foundation, on the other hand, recommend the use of low-dose aspirin for the primary prevention of CVD in individuals with diabetes, but only if the risk of CVD and bleeding is minimal (16). These recommendations were based on a pooled analysis of nine studies, which revealed a moderate reduction in the risk of cardiovascular events using aspirin (although without a precise assessment of the effect size). The authors of the recommendation mention current investigations that will bring - new information in this area, given the - debatable effect of aspirin in primary CVD prevention in adults with diabetes.

The randomized experiment ASCEND (Study of Cardiovascular Events in Diabetes) provides encouraging results on the effects of low-dose aspirin for the prevention of serious vascular events in adults with diabetes; however, the results of this trial was not encouraging as an increased risk of gastrointestinal and extracranial bleeding events were reported in the aspirin group compared to placebo (6).

*Rationale:* We conducted an updated systematic meta-analysis to address the benefits and hazards of aspirin for the primary prevention of CVD, all-cause mortality events, and CV events in persons with diabetes, given the significant clinical interest in the subject and conflicting results.

*Objective:* To evaluate the effectiveness of aspirin compared to control (placebo or no treatment) for prevention of CVD and all-cause mortality events and adverse CV events in persons with diabetes.

## Materials And Methods

We followed the **PRISMA** statement (Preferred Reporting Items for Systematic Reviews and Meta-Analyses) normative recommendations in this study with the registration number XU#/IRB/2020/1005.

### Data Sources and Strategy for Search

Two separate authors used MEDLINE (via PubMed), CINAHL (via Ebsco), Scopus, and Web of Sciences, and the Cochrane electronic databases to conduct a duplicate search for randomized controlled trials published until December 2020 (date last searched). The computer-assisted searches included words related to using the predefined search terms aspirin, cardiovascular disease (CVD), diabetes, efficacy, OR meta-analysis.

According to the selection criteria, two independent reviewers assessed the titles and abstracts of all initially recognized papers. Full texts were collected from papers that met all the criteria for inclusion. Additional papers were found by searching the reference lists of chosen research and relevant reviews on the topic.

### Selection and Qualifying Criteria for Studies

Intervention studies with data on the use of aspirin for the primary prevention of CVD in patients with diabetes and data on several cardiovascular and all-cause mortality endpoints were sought. Intervention studies that were randomized controlled, open, or blinded trials were eligible if 1) aspirin was compared with placebo or no treatment in patients with or without diabetes, and without a history or clinical evidence of CVD; 2) screened for the eligibility, the studies had to be controlled (placebo or control group) but could be open-label or blinded; 3) patients above 18 years of age either who have diabetes was included, or full text was acquired for further evaluation if the citation was deemed pertinent; 4) had at least a 12month follow-up period.

Non-randomized studies comparing aspirin to another antiplatelet drug, studies including persons with known CVD, and secondary publications of trials previously included in the analysis were excluded.

### Data Extraction

Two separate writers (HM and QG) extracted data, and in the event of any inconsistencies, a consensus was obtained with the help of a third and fourth author (HN and XL). In order to collect the pertinent data, a pre-designed data extraction form was employed. The data extracted from the selected trials included appropriate study-level information, number of diabetic participants, age group, intervention, control, and follow-up time.

The outcomes included all-cause mortality, composite outcomes of major adverse cardiovascular events (MACE)-cardiovascular mortality, non-fatal myocardial infarction and stroke, and bleeding events.

### Risk of Bias *Evaluation*

The Cochrane Collaboration’s risk of bias tool was used to assess the methodological quality of the included studies (21). The tool includes the following criteria: randomization, allocation concealment, blinding, and completeness of follow-up. The risk of bias for each item was graded as high, low, or unclear risk based on the authors’ judgment.

### Quantitative Data Synthesis

The outcomes were treated as dichotomous data, and the odds ratio (OR) and 95% confidence interval (95% CI) were used to estimate the pooled results from the studies. Meta-analysis was performed using Review Manager (RevMan Version 5. Copenhagen: The Nordic Cochrane Center, The Cochrane Collaboration. 2020). Meta-analyses were done using a random-effects model (Mantel-Haenszel method), and heterogeneity between the included studies was evaluated using the *I^2^* statistic with small heterogeneity for *I^2^* values up to 25%, moderate heterogeneity for *I^2^* values between 25% and 50%, and high heterogeneity for *I^2^* values > 50% (22). The random-effects model was chosen to account for differences in characteristics between the studies, particularly participants’ age, type of control (placebo or no treatment), and region of trial.

## Results

### Literature Search Results

**Table 1** summarizes the characteristics of the studies included in the meta-analysis, which shows differences in demographic characteristics (age, gender, CVD risk factors), follow-up time ranging from 3.6-10.1 years, aspirin dose and comparators between the different trials

**Table 1:** Characteristics of included studies.

Study name	No. of diabetic participants	Demographic characteristics	Intervention	Control	Follow-up (years)
ASCEND 2018 (6)	15,480	Diabetic men and women, > 40 years, diabetes mellitus with no known CV condition	Aspirin (100 mg/day)	Placebo	7.5
ASPREE 2018 (7)	2,057	Men and women, > 70 years, No coronary heart disease, cerebrovascular disease and atrial fibrillation	Aspirin (100 mg/day)	Placebo	4.7
ETDRS 1992 (8)	3,711	Men and women with type 1 and type 2 diabetes, 18-70 years	Aspirin (650 mg/day)	Placebo	5
JPAD 2008 (9)	2,539	Men and women with type 2 diabetes, 30-85 years	Aspirin (81-100 mg/day)	No aspirin	4.37
JPPP 2014 (10)	4,903	Men and women, with diabetes mellitus and dyslipidaemia, 60-85 years, no atherosclerotic disease	Aspirin (100 mg/day)	No aspirin	5.02
POPADAD 2008 (11)	1,276	Men and women with type 1 or type 2 diabetes mellitus, > 40 years, asymptomatic peripheral disease	Aspirin + antioxidant Aspirin+placebo (100 mg/day)	Placebo	6.7
PPP 2003 (12)	1,031	Men and women with diabetes mellitus and hypertension, hypercholesterolemia > 50 years	Aspirin (100 mg/day)	Vitamin E (300 mg/day)	3.6
WHS 2005 (13)	1,027	Women > 45 years, no history of coronary heart diseases, cerebrovascular disease or cancer	Aspirin (100 mg on alternate days)	Placebo	10.1

**Table 2** summarizes the outcomes for all-cause mortality, cardiovascular mortality, MACE, and bleeding events in the studies included for meta-analysis where significant differences between the aspirin and control group for a particular outcome have been indicated. Percentage of patients in each group developing an outcome does not seem to be significantly different for most trials

**Table 2:** Outcomes of all-cause mortality, cardiovascular mortality, composite outcome of major adverse cardiovascular events (MACE) and bleeding events in the included studies.

Study name	All-cause mortality (n/N)	Cardiovascular mortality (n/N)	MACE (n/N)	Bleeding events (n/N)
ASCEND 2018 (6)	Aspirin: 748/7740 (9.66%) Placebo: 792/7740 (10.23%)	Aspirin: 210/7740 (2.71%) Placebo: 226/7740 (2.92%)	Aspirin: 658/7740 (8.50%) Placebo: 743/7740 (9.60%)	Aspirin: 314/7740 (4.06%) Placebo: 245/7740 (3.17%)
ASPREE 2018 (7)	Aspirin: 87/1027 (8.47%) Placebo: 68/1030 (6.60%)	–	Aspirin: 54/1027 (5.26%) Placebo: 55/1030 (5.34%)	–
ETDRS 1992 (8)	Aspirin: 340/1856 (18.32%) Placebo: 366/1855 (19.73%)	Aspirin: 244/1856 (13.15%) Placebo: 275/1855 (14.82%)	Aspirin: 350/1856 (18.86%) Placebo: 379/1855 (20.43%)	–
JPAD 2008 (9)	Aspirin: 34/1262 (2.69%) Placebo: 38/1277 (2.98%)	Aspirin: 1/1262 * (0.08%) Placebo: 10/1277* (0.78%)	Aspirin: 68/1262 (5.39%) Placebo: 86/1277 (6.73%)	Aspirin: 34/1262 * (2.69%) Placebo: 12/1277* (0.94%)
JPPP 2014 (10)	–	–	Aspirin: 86/2445 (3.52%) Placebo: 98/2458 (3.99%)	–
POPADAD 2008 (11)	Aspirin: 94/638 (14.73%) Placebo: 101/638 (15.83%)	Aspirin: 43/638 (6.74%) Placebo: 35/638 (5.49%)	Aspirin: 105/638 (16.46%) Placebo: 108/638 (16.93%)	Aspirin: 28/638 (4.39%) Placebo: 31/638 (4.86%)
PPP 2003 (12)	Aspirin: 25/519 (4.82%) Placebo: 20/512 (3.90%)	Aspirin: 10/519 (1.93%) Placebo: 8/512 (1.56%)	Aspirin: 20/519 (3.85%) Placebo: 22/512 (4.30%)	–
WHS 2005 (13)	–	–	Aspirin: 58/514 (11.28%) Placebo: 62/513 (12.09%)	–

n/N represents no. of events or outcomes in the group divided by the total no. of participants in each trial group

*represents a significant difference between the two groups for the particular outcome.

The present meta-analysis comprised 32,024 -patients with diabetes; 16,001 allocated to aspirin and 16,023 allocated to the control group.

### Risk of Bias Assessment

**Figure 1:**
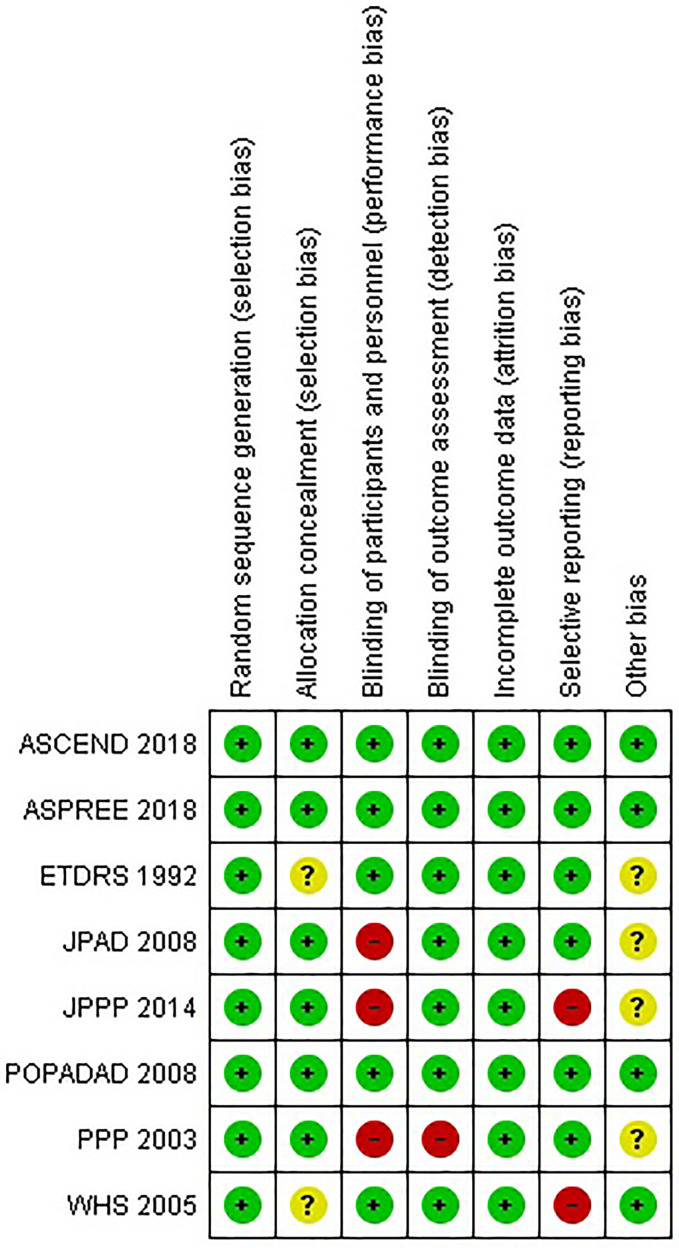
Risk of bias summary for studies included in the meta-analysis.

According to the Cochrane Collaboration’s risk of bias tool, all included experiments had a low chance of bias (**Figure 1**).

### Meta-Analysis Results

**Figure 2:**
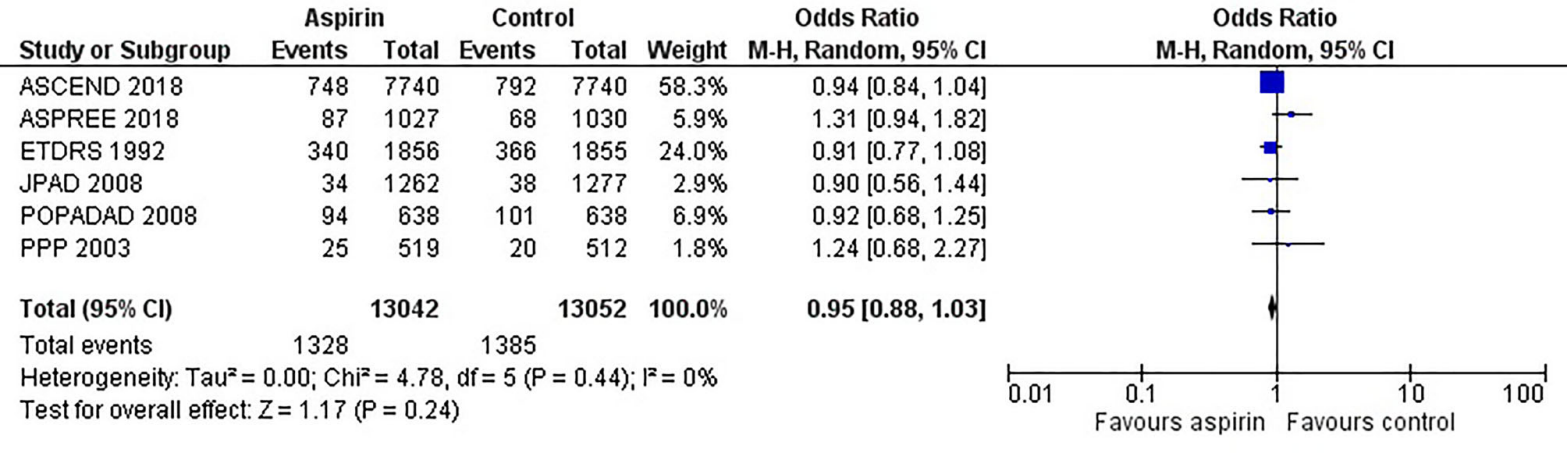
Forest plot for the outcome of all-cause mortality using a random-effects model. Odds ratios and 95% confidence intervals are shown. Control: placebo or no treatment.

**Figure 3:**
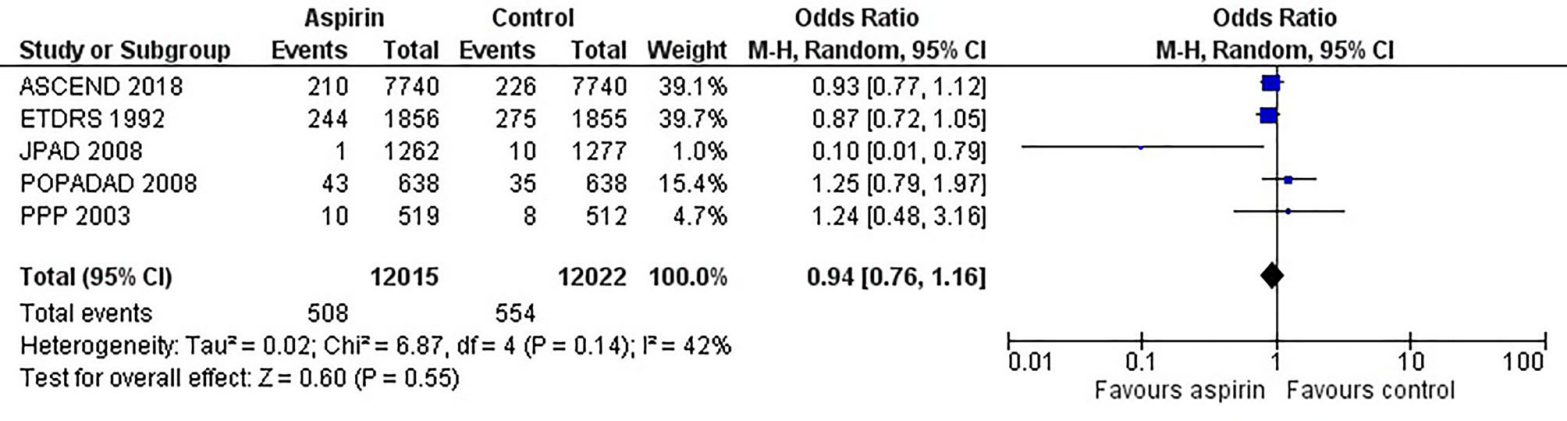
Forest plot for the outcome of cardiovascular mortality using a random-effects model. Odds ratios and 95% confidence intervals are shown. Control: placebo or no treatment.

**Figure 4:**
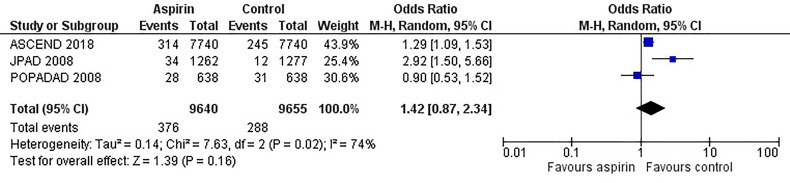
Forest plot for the outcome of bleeding events using a random-effects model. Odds ratios and 95% confidence intervals are shown. Control: placebo or no treatment.

**Figure 5:**
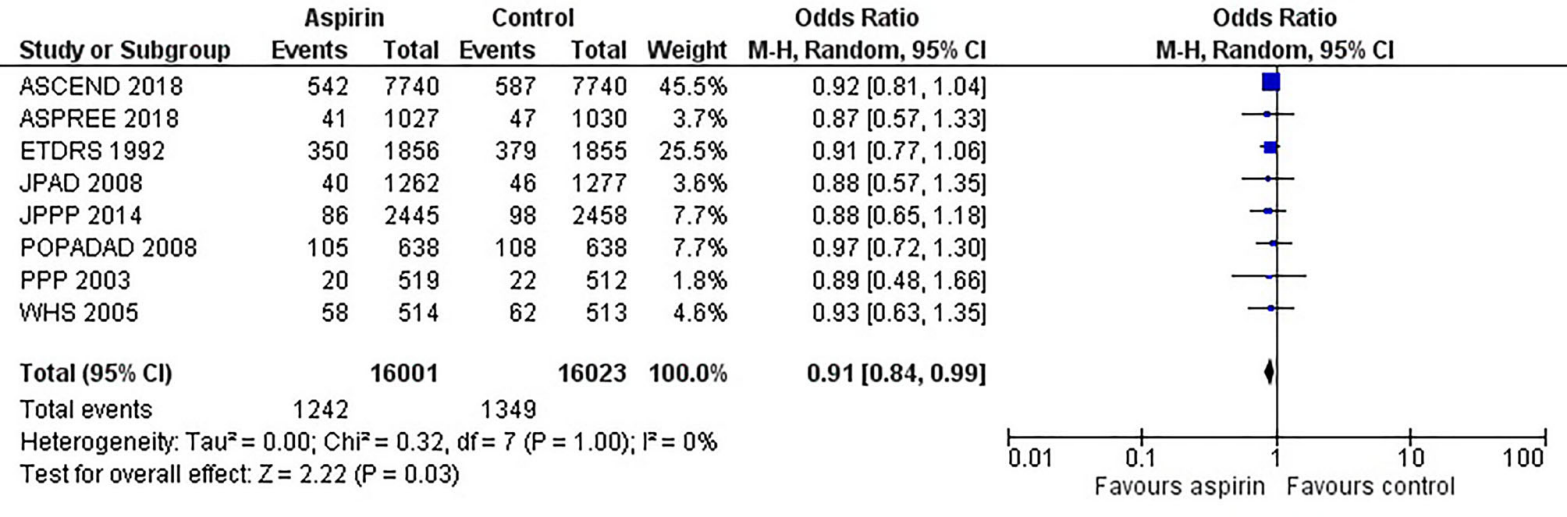
Forest plot for the outcome of composite of major adverse cardiovascular events (MACE) using a random-effects model. Odds ratios and 95% confidence intervals are shown. Control: placebo or no treatment.

**Figure 2** is the forest plot of the odds ratio of all-cause mortality in patients with diabetes in the aspirin or control group using the random-effects model. Compared with the control group, aspirin did not significantly reduce the risk of all-cause mortality (OR 0.95, 0.88-1.03, P=0.24, *I^2^ = *0%). Similarly, aspirin did not decrease the risk of cardiovascular mortality (OR 0.94, 0.76-1.16, P=0.55, *I^2^ = *42%) or increase the risk of bleeding events (OR 1.42, 0.87-2.34, P=0.16, *I^2^ = *74%) compared to the control group (**Figures 3** and **4**). In contrast, the risk of MACE was significantly decreased upon aspirin administration compared to control (OR 0.89, 0.82-0.96, P=0.003, *I^2^ = *0%) (**Figure 5**). The *I^2^* values indicative of heterogeneity varied from low to high for the different outcomes.

## Discussion

Aspirin, an antithrombotic agent, is postulated to decrease the risk of cardiovascular death and events in patients with prior cardiovascular disease and is a mainstay for secondary prevention (23–26). However, the role of aspirin in the primary prevention of cardiovascular events is debatable. Myocardial infarction and ischemic stroke are the leading causes of morbidity and mortality in patients with type 2 diabetes due to target organ damage, atherosclerosis, increased platelet turnover and reactivity, and higher thrombin generation (27–29). Therefore, it is expected that aspirin treatment would be beneficial in this diabetic population. Furthermore, ESC 2019 guidelines have recommended the use of aspirin (75-100 mg/day) for the primary prevention of cardiovascular diseases in patients with diabetes mellitus at high risk in the absence of clear contraindications (30). Despite this, aspirin is limited based on an increased risk of bleeding, making it necessary to understand the risk-benefit profile when using aspirin for primary prevention of cardiovascular events.

The current meta-analysis showed that aspirin did not decrease the risk of all-cause mortality, cardiovascular mortality and did not increase the risk of adverse events such as bleeding in diabetics without prior cardiovascular diseases, according to previous meta-analyses (31–33). The absence of a significant reduction in all-cause mortality and cardiovascular mortality in the diabetic population can be attributed to aspirin resistance. People with diabetes have altered platelet function, abnormalities in endothelial and vascular smooth muscle function, increased production of pro-inflammatory markers and clotting factors, and hyperglycemia which can all affect the functional benefits of aspirin.

The studies included in the current meta-analysis were heterogeneous with respect to patient population, especially in terms of age and CV risk factors. POPADAD was a trial that randomized patients with type I or type II diabetes and ankle-brachial pressure index of < 0.99 (an indicator of peripheral artery disease) but no symptomatic CVD. In this trial, no differences were found between aspirin and placebo group in terms of mortality or MACE. In contrast, in the JPAD trial that included patients with diabetes with no history of atherosclerotic disease, aspirin was found to cause a significant decrease in CV mortality. However, this beneficial effect was associated with a significantly higher bleeding risk associated with aspirin use. The PPP and PPP trials enrolled patients with CV risk factors (hypercholesterolemia, hypertension, and dyslipidemia). In these two trials, no difference was found between mortality and MACE outcomes between aspirin and control groups. These findings suggest a difference in effect of aspirin based on baseline CVD risk. Gelbenegger et al. demonstrated a gender difference in aspirin effects in their meta-analysis (32). A significant reduction in MACE was observed in males following aspirin administration compared to females.

However, aspirin was found to significant reduction in MACE; the risk of MACE was 12% higher in the control group than in the aspirin group. This beneficial effect resulted from a pooled analysis of 8 studies with higher events rates and sufficient power to detect a significant reduction in MACE. In addition, there was no heterogeneity (*I^2^ = *0%) between the studies for the outcome of MACE, suggesting consistent definitions for MACE were used between studies and indicates the reliability of the pooled estimates and a clear beneficial effect of aspirin in accordance with ADA and AHA guidelines Furthermore, the risk of bias in the included studies is low ensuring reliability in the meta-analysis results (34–36).

When considering the point estimate for the odds ratio of bleeding events, the odds of bleeding were 42% higher in the aspirin group than control, although the overall result was not statistically significant. A limited number of studies have reported bleeding outcomes (*n=3* studies), making it difficult to conclude the risks and benefits associated with aspirin use. A higher risk of gastrointestinal bleeding has been demonstrated in people with low-dose and long-term aspirin treatment, elderly patients, and those with lower cardiovascular risk (34-36). However, the current studies were not sufficiently powered to detect these differences, resulting in imprecise estimates. Furthermore, the definition of bleeding is inconsistent between the limited studies. Some studies have reported a composite of major bleeding events such as intracranial hemorrhage and gastrointestinal bleeding (ASCEND and JPAD trials), whereas only gastrointestinal bleeding (POPADAD trial) has been reported in one study, and this could have led to the high heterogeneity (*I^2^ = *74%) in the meta-analysis of the bleeding outcomes. High heterogeneity values for cardiovascular mortality outcomes can be potentially associated with the inclusion of older studies (ETDRS 1992 and PPP 2003) in which medical care, treatment protocols, and early diagnostic services were considerably different. Differences in follow-up time between the studies, control group (placebo or no aspirin), and age of the participants can also contribute to heterogeneity.

Overall, this work provides a current and comprehensive meta-analysis to assess the benefits and risks associated with aspirin use for primary prevention of cardiovascular events in diabetic participants. However, this work is limited by the number of published studies available and the inability to perform subgroup analyses and obtain reliable estimates for pooled effects. Furthermore, heterogeneous definitions for endpoints, particularly bleeding events, and lack of availability of this data from the clinical studies can lead to biased estimates and selective reporting bias, which is seen in the risk of bias analysis. In addition, the inclusion of older studies published more than 20 years ago may skew the pooled results based on changing treatment guidelines. Therefore, it is crucial to interpret the results cautiously and conduct more studies in which the event rate is higher to obtain precise estimates of pooled effects, particularly for events such as bleeding.

## Conclusion

Although aspirin use was not found to be associated with a decrease in all-cause and CV mortality in patients with diabetes, a clear beneficial effect was seen of aspirin in decreasing the incidence of MACE. Furthermore, the risk of bleeding was not increased following aspirin use, suggesting a beneficial effect of aspirin in the primary prevention of cardiovascular events in patients with diabetes. The results of this meta-analysis are promising as they include diverse patient group in terms of age and risk factors.

## Data Availability Statement 

The raw data supporting the conclusions of this article will be made available by the authors, without undue reservation.

## Author Contributions 

HM: has designed the concept. QG, Data acquisition. HN drafted the manuscript. XL literature search. RW Final proofreading and editing. All authors contributed to the article and approved the submitted version.

## Conflict of Interest

The authors declare that the research was conducted in the absence of any commercial or financial relationships that could be construed as a potential conflict of interest.

## Publisher’s Note

All claims expressed in this article are solely those of the authors and do not necessarily represent those of their affiliated organizations, or those of the publisher, the editors and the reviewers. Any product that may be evaluated in this article, or claim that may be made by its manufacturer, is not guaranteed or endorsed by the publisher.

## References

[1] Piepoli MF Hoes AW Agewall S Albus C Brotons C Catapano AL . ESC Scientific Document Group. 2016 European Guidelines on Cardiovascular Disease Prevention in Clinical Practice: The Sixth Joint Task Force of the European Society of Cardiology and Other Societies on Cardiovascular Disease Prevention in Clinical Practice (Constituted by Representatives of 10 Societies and by Invited Experts) Developed With the Special Contribution of the European Association for Cardiovascular Prevention & Rehabilitation (EACPR). Eur Heart J (2016) Aug 1 37(29):2315–81. doi: 10.1093/eurheartj/ehw106 PMC4986030 27222591

[2] Bulugahapitiya U Siyambalapitiya S Sithole J Idris I . Is Diabetes a Coronary Risk Equivalent? Systematic Review and Meta-Analysis. Diabetes Med (2009) 26:142–8. doi: 10.1111/j.1464-5491.2008.02640.x 19236616

[3] Hennekens CH Dyken ML Fuster V . Aspirin as a Therapeutic Agent in Cardiovascular Disease: A Statement for Healthcare Professionals From the American Heart Association. Circulation (1997) 96:2751–3. doi: 10.1161/01.CIR.96.8.2751 9355934

[4] Ajani UA Ford ES Greenland KJ Giles WH Mokdad AH . Aspirin Use Among U.S. Adults: Behavioral Risk Factor Surveillance System. Am J Prev Med (2006) 30:74–7. doi: 10.1016/j.amepre.2005.08.042 16414427

[5] Ittaman SV VanWormer JJ Rezkalla SH . The Role of Aspirin in the Prevention of Cardiovascular Disease. Clin Med Res (2014) 12:147–54. doi: 10.3121/cmr.2013.1197 PMC4317158 24573704

[6] ASCEND Study Collaborative Group Bowman L Mafham M Wallendszus K Stevens W Buck G Barton J . Effects of Aspirin for Primary Prevention in Persons With Diabetes Mellitus. The ASCEND Study Collaborative Group. N Engl J Med (2018) 379(16):1529–39. doi: 10.1056/NEJMoa1804988 30146931

[7] McNeil JJ Nelson MR Woods RL Lockery JE Wolfe R Reid CM . ASPREE Investigator Group. Effect of Aspirin on All-Cause Mortality in the Healthy Elderly. N Engl J Med (2018) 379(16):1519–28. doi: 10.1056/NEJMoa1803955 PMC6433466 30221595

[8] ETDRS Investigators . Aspirin Effects on Mortality and Morbidity in Patients With Diabetes Mellitus. JAMA (1992) 268(10):1292–300. 10.1001/jama.1992.03490100090033 1507375

[9] Ogawa H Nakayama M Morimoto T Uemura S Kanauchi M Doi N . Japanese Primary Prevention of Atherosclerosis With Aspirin for Diabetes (JPAD) Trial Investigators. Low-Dose Aspirin for Primary Prevention of Atherosclerotic Events in Patients With Type 2 Diabetes. A Randomized Controlled Trial. JAMA (2008) 300(18):2134–41. doi: 10.1001/jama.2008.623 18997198

[10] Teramoto T Shimada K Uchiyama S Sugawara M Goto Y Yamada N . Rationale, Design, and Baseline Data of the Japanese Primary Prevention Project (PPP)-A Randomized, Open-Label, Controlled Trial of Aspirin Versus No Aspirin in Patients With Multiple Risk Factors for Vascular Events. Am Heart J (2010) 159(3):361–369.e4. doi: 10.1016/j.ahj.2009.11.030 20211296

[11] Belch J MacCuish A Campbell I Cobbe S Taylor R Prescott R . The Prevention of Progression of Arterial Disease and Diabetes (POPADAD) Trial: Factorial Randomised Placebo-Controlled Trial of Aspirin and Antioxidants in Patients With Diabetes and Asymptomatic Peripheral Arterial Disease. BMJ (2008) 337:a1840. doi: 10.1136/BMJ.a1840 18927173 PMC2658865

[12] Sacco M Pellegrini F Roncaglioni MC Avanzini F Tognoni G Nicolucci A . Primary Prevention of Cardiovascular Events With Low-Dose Aspirin and Vitamin E in Type 2 Diabetic Patients. Results of the Primary Prevention Project (PPP) Trial. Diabetes Care (2003) 26(12):3264–72. doi: 10.2337/diacare.26.12.3264 14633812

[13] Ridker PM Cook NR Lee IM Gordon D Gaziano JM Manson JE . A Randomized Trial of Low-Dose Aspirin in the Primary Prevention of Cardiovascular Disease in Women. N Eng J Med (2005) 352:1293–304. doi: 10.1056/NEJMoa050613 15753114

[14] Preventive Services Task Force US . Aspirin for the Primary Prevention of Cardiovascular Events: Recommendation and Rationale. Ann Intern Med (2002) 136:157–60. doi: 10.7326/0003-4819-136-2-200201150-00015 11790071

[15] Mahmoud AN Gad MM Elgendy AY Bavry AA . Efficacy and Safety of Aspirin for Primary Prevention of Cardiovascular Events: A Meta-Analysis and Trial Sequential Analysis of Randomized Controlled Trials. Eur Heart J (2019) Feb 14 40(7):607–17. doi: 10.1093/eurheartj/ehy813 30561620

[16] Colwell JA . American Diabetes Association. Aspirin Ther diabetes Diabetes Care (2003) 26:S87–8. 10.2337/diacare.26.2007.s87 12502626

[17] Steering Committee of the Physicians’ Health Study Research Group. Final Report on the Aspirin Component of the Ongoing Physicians’ Health Study. N Engl J Med (1989) 321:129–35. doi: 10.1056/NEJM198907203210301 2664509

[18] The Medical Research Council’s General Practice Research Framework. Thrombosis Prevention Trial: Randomised Trial of Low-Intensity Oral Anticoagulation With Warfarin and Low-Dose Aspirin in the Primary Prevention of Ischaemic Heart Disease in Men at Increased Risk. Lancet (1998) 351:233–41. doi: 10.1016/S0140-6736(97)11475-1 9457092

[19] Hansson L Zanchetti A Carruthers SG Dahlöf B Elmfeldt D Julius S . Effects of Intensive Blood-Pressure Lowering and Low-Dose Aspirin in Patients With Hypertension: Principal Results of the Hypertension Optimal Treatment (HOT) Randomised Trial. Lancet (1998) 351:1755–62. doi: 10.1016/S0140-6736(98)04311-6 9635947

[20] Pignone M Alberts MJ Colwell JA Cushman M Inzucchi SE Mukherjee D . Aspirin for Primary Prevention of Cardiovascular Events in People With Diabetes. J Am Coll Cardiol (2010) 55:2878–86. doi: 10.1016/j.jacc.2010.04.003 20579547

[21] Higgins JPT Altman DG Gøtzsche PC Jüni P Moher D Oxman AD . The Cochrane Collaboration’s Tool for Assessing Risk of Bias in Randomised Trials. BMJ (2011) 343:d5928. doi: 10.1136/bmj.d5928 22008217 PMC3196245

[22] Higgins JPT Thompson SG Deeks JJ Altman D . Measuring Inconsistency in Meta-Analyses. BMJ (2003) 327:557–60. doi: 10.1136/bmj.327.7414.557 PMC192859 12958120

[23] Fox CS Coady S Sorlie PD D'Agostino RB Sr Pencina MJ Vasan RS . Increasing Cardiovascular Disease Burden Due to Diabetes Mellitus: The Framingham Heart Study. Circulation (2007) 115(12):1544–50. doi: 10.1161/CIRCULATIONAHA.106.658948 17353438

[24] Tierney Fennessy F Hayes DB . ABC of Arterial and Vascular Disease: Secondary Prevention of Peripheral Vascular Disease. BMJ (2000) 320:1262–5. doi: 10.1136/bmj.320.7244.1262 PMC1117996 10797042

[25] Belch JJ Topol EJ Agnelli G Bertrand M Califf RM Clement DL . Critical Issues in Peripheral Arterial Disease Detection and Management. A call to action Arch Intern Med (2003) 163:884–92. doi: 10.1001/archinte.163.8.884 12719196

[26] Scottish Intercollegiate Guidelines Network . Diagnosis and Management of Peripheral Arterial Disease: A National Clinical Guideline (2006). Available at: www.sign.ac.uk/pdf/sign89.pdf.

[27] Creager MA Luscher TF Cosentino F Beckman JA . Diabetes and Vascular Disease: Pathophysiology, Clinical Consequences, and Medical Therapy: Part I. Circulation (2003) 108:1527–32. doi: 10.1161/01.CIR.0000091257.27563.32 14504252

[28] Varo N Vicent D Libby P Nuzzo R Calle-Pascual AL Bernal MR . Elevated Plasma Levels of the Atherogenic Mediator Soluble CD40 Ligand in Diabetic Patients: A Novel Target of Thiazolidinediones. Circulation (2003) 107:2664–9. doi: 10.1161/01.CIR.0000074043.46437.44 12742991

[29] Evangelista V Totani L Rotondo S Lorenzetti R Tognoni G De Berardis G . Prevention of Cardiovascular Disease in Type-2 Diabetes: How to Improve the Clinical Efficacy of Aspirin. Thromb Haemost (2005) 93:8–16. doi: 10.1160/TH04-07-0453 15630484

[30] Cosentino F Grant PJ Aboyans V Bailey CJ Ceriello A Delgado V . 2019 ESC Guidelines on Diabetes, Pre-Diabetes, and Cardiovascular Diseases Developed in Collaboration With the EASD. Eur Heart J (2020) 41:255–323. doi: 10.1093/eurheartj/ehz486 31497854

[31] Caldeira D Alves M David C Costa J Ferreira JJ Pinto FJ . Aspirin in the Primary Prevention of Cardiovascular Disease on Diabetic Patients: Systematic Review and Meta-Analysis. Prim Care Diabetes (2020) 14(3):213–21. doi: 10.1016/j.pcd.2019.11.004 31791903

[32] Gelbenegger G Postula M Pecen L Halvorsen S Lesiak M Schoergenhofer C . Aspirin for the Primary Prevention of Cardiovascular Disease: A Meta-Analysis With a Particular Focus on Subgroups. BMC Med (2019) 17:198. doi: 10.1186/s12916-019-1428-0 31679516 PMC6827248

[33] Kunotsor SK Seidu S Khunti K . Aspirin For the Primary Prevention of Cardiovascular and All-Cause Mortality Events in Diabetes: Updated Meta-Analysis of Randomized Controlled Trials. Diabetes Med (2017) 34(3):316–27. doi: 10.1111/dme.13133 27086572

[34] Patrono C Rodríguez LA Landolfi R Baigent C . Low-Dose Aspirin for the Prevention of Atherothrombosis. N Eng J Med (2005) 353(22):2373–83. doi: 10.1056/NEJMra052717 16319386

[35] Derry S Loke YK . Risk of Gastrointestinal Haemorrhage With Long Term Use of Aspirin: Meta-Analysis. BMJ (2000) 321(7270):1183–7. doi: 10.1136/BMJ.321.7270.1183 PMC27521 11073508

[36] Hernández-Díaz S García Rodríguez LA . Cardioprotective Aspirin Users and Their Excess Risk of Upper Gastrointestinal Complications. BMC Med (2006) 4:22. doi: 10.1186/1741-7015-4-22 16987411 PMC1590044

